# Effect of Ma'aljobon (Whey) Consumption on Functional Constipation in Older Adults: A Randomized Double‐Blind Controlled Trial

**DOI:** 10.1002/hsr2.71881

**Published:** 2026-07-08

**Authors:** Farzaneh Saadat Talab, Mehrdad Karimi, Hadi Najafi, Atefe Esmailpour Moalem, Shahab Papi

**Affiliations:** ^1^ Department of Geriatric Health University of Social Welfare and Rehabilitation Sciences Tehran Iran; ^2^ Department of Traditional Medicine Tehran University of Medical Sciences Tehran Iran; ^3^ PhD Student in Gerontology, Department of Geriatric Health, Faculty of Health Sciences Tabriz University of Medical Sciences Tabriz Iran; ^4^ Department of Geriatric Nursing, School of Nursing and Midwifery Mazandaran University of Medical Sciences Sari Iran; ^5^ Social Determinants of Health Research Center, School of Nursing and Midwifery Lorestan University of Medical Sciences Khorramabad Iran

**Keywords:** clinical trial, functional constipation, Iranian traditional medicine, Ma'aljobon, whey protein

## Abstract

**Background and Aims:**

Constipation is one of the most common gastrointestinal disorders worldwide, and its prevalence increases with age. The aim of the present study was to evaluate the effect of Ma'aljobon (a type of whey) on functional constipation (FC) in older adults.

**Methods:**

This study was a randomized, double‐blind, placebo‐controlled, parallel‐group, phase III trial. A total of 100 older adults (aged 60–90 years; 73.4% female) with FC, diagnosed according to the Rome III criteria, were recruited from a healthcare center for a trial conducted at a traditional medicine clinic in Sabzevar, Iran, using a convenience sampling method. Participants were randomly assigned to receive either 15 g of Ma'aljobon powder (*n *= 50) or a matching placebo (*n* = 50) daily for 4 weeks. The primary outcome was the change in stool consistency, assessed with the Bristol Stool Form Scale. The trial was registered with the Iranian Registry of Clinical Trials (No. IRCT20180624040216N1).

**Results:**

At baseline, there were no significant differences between the groups (all *p* > 0.05). After 4 weeks, the Ma'aljobon group showed statistically significant improvements in all secondary FC parameters compared to the placebo group (all *p* < 0.001). For the primary outcome of stool consistency, the odds of having a normal versus hard stool were significantly higher in the Ma'aljobon group than in the placebo group (odds ratio = 39.1, 95% CI [2.3, 670.3]; *p* < 0.001).

**Conclusion:**

The results of this trial indicate that Ma'aljobon has a significant impact on alleviating the symptoms of FC in older adults and is a well‐tolerated intervention.

## 1 Background

The global population is aging at an unprecedented rate [1]. The number of individuals aged 60 and above is projected to increase from 841 million in 2013 to over 2 billion by 2050 [2]. Functional constipation (FC) is one of the most common gastrointestinal disorders worldwide, and its prevalence increases with age, affecting 33.5% of older adults [3, 4]. In Iran, a recent review reported a national prevalence of FC ranging from 1.4% to 3% [5].

While constipation is broadly characterized by difficult, infrequent, or incomplete defecation [6], FC is specifically defined by the Rome III criteria. These criteria include a combination of objective indicators—such as reduced frequency of bowel movements, hard or lumpy stools, and the need for manual maneuvers to facilitate defecation—and subjective symptoms, including straining, a sensation of anorectal obstruction, and a feeling of incomplete evacuation, all observed over a defined period [7].

The etiology of constipation in older adults is multifactorial, often resulting from a combination of age‐related decline in gastrointestinal motility, insufficient fiber and fluid intake, neurodegenerative conditions like Parkinson's disease and dementia, neurological events such as stroke or spinal cord injury, metabolic conditions such as hypoglycemia or hyperglycemia, and certain medications [8]. Furthermore, conditions like angina pectoris, rectal prolapse, and hemorrhoids can contribute to or exacerbate constipation [8]. This disorder significantly impairs quality of life and can lead to complications such as depression, irritability, headache, loss of appetite, and anal fissures [9, 10].

While pharmacological treatments—such as bulk‐forming agents, osmotic and stimulant laxatives, and stool softeners—are commonly used, they often produce undesirable side effects, making them unsuitable for some older adults with comorbidities [11]. Patient satisfaction with these treatments can be low, with up to 47% of users reporting dissatisfaction due to inconsistent efficacy, safety concerns, unpleasant taste, and cost [12]. Consequently, there is increasing interest in non‐pharmacological, safe, and affordable alternatives for managing constipation in older populations.

Iranian Traditional Medicine (ITM), also referred to as Persian or Unani Medicine, is a comprehensive system rooted in centuries of medical practice and philosophical theory [13]. Among the therapeutic substances described in ITM, Ma'aljobon (a type of whey) is widely used. Derived from the cheese‐making process [14], whey proteins represent 15%–20% of total milk protein and primarily include β‐lactoglobulin (35%–65%), α‐lactalbumin (12%–25%), immunoglobulins (8%), albumin (5%), and lactoferrin (1%) [14, 15]. Whey is considered nutritionally beneficial both as a standalone supplement and in combination with other therapies [16]. It is also easier to digest than many other protein sources [17]. In ITM, Ma'aljobon is used for a range of conditions—including stroke, insomnia, attention deficit hyperactivity disorder (ADHD), headaches, joint diseases, reduction in fasting blood glucose levels in diabetic patients, primary hypertension, hemorrhoids, and digestive issues—some of which are now supported by emerging scientific evidence [16, 18, 19, 20, 21].

Beyond its traditional use, modern studies suggest that whey and its components may benefit gastrointestinal health through several mechanisms. Its high lactose content exerts an osmotic effect, drawing water into the intestines and softening stool [22]. Whey proteins—especially lactoferrin and α‐lactalbumin—have prebiotic effects, promoting the growth of beneficial bacteria like *Bifidobacterium* and *Lactobacillus* [23]. These components also enhance short‐chain fatty acids (SCFAs) production and lower colonic pH, processes known to relieve constipation [24]. Such effects are particularly relevant in older adults due to age‐related declines in microbial diversity. While studies in infants show improved stool consistency with whey‐based formulas [25], clinical evidence in geriatric populations remains scarce. Addressing this gap, the present study evaluates the therapeutic potential of Ma'aljobon in treating FC among older adults.

## 2 Materials and Methods

### 2.1 Study Design

This study was a randomized, double‐blind, placebo‐controlled, parallel‐group, phase III clinical trial conducted over 4 weeks. Participants were recruited from a healthcare center in Sabzevar, Iran, while the screening and all intervention procedures were conducted at a traditional medicine clinic. The trial was designed to test the hypothesis that Ma'aljobon would improve the weekly frequency of bowel movements and stool consistency more effectively than a placebo. The study protocol adhered to the CONSORT 2010 Statement.

The study involved five visits: an initial screening visit, a baseline visit (Day 0), and three follow‐up visits at the end of weeks 1 (Day 7), 2 (Day 14), and 3 (Day 21), with the final assessment at week 4 (Day 28). At the screening visit, a comprehensive medical history was taken, concomitant medications were recorded, and eligibility against inclusion/exclusion criteria was assessed. Following baseline confirmation and a physical examination, eligible participants completed several questionnaires. Throughout the 4‐week intervention, data on stool characteristics, treatment compliance, and any adverse events were collected weekly. Adverse events were graded using the Common Terminology Criteria for Adverse Events (CTCAE v4.0).

### 2.2 Sample Size

The sample size was calculated based on the expected change in the number of bowel movements with normal consistency (defined as types 3 or 4 on the Bristol Stool Form Scale) per week, a key variable directly related to the primary outcome. Based on a preliminary pilot study, we anticipated a mean increase of 1.5 normal bowel movements per week in the intervention group compared to the placebo group, with a pooled standard deviation of 2.5. With a two‐sided significance level (*α*) set at 0.05 and a desired statistical power (1 −* β*) of 80%, the calculation indicated that a minimum of 44 participants per group was required. To account for an estimated 10% dropout rate, we aimed to recruit 50 participants for each arm of the study, for a total of 100 participants.

### 2.3 Inclusion and Exclusion Criteria

Eligible participants were required to be 60 years of age or older, mentally alert, and capable of completing questionnaires. They must have had no history of gastrointestinal surgery, obstructive or inflammatory gastrointestinal diseases, or renal failure, and must not have undergone any major surgery within the last 6 months. Conversely, exclusion criteria included any unwillingness to continue the intervention; significant dietary changes made to relieve constipation during the trial period; planned major lifestyle modifications (e.g., in diet, exercise, or travel); regular use of medications or dietary supplements known to cause constipation; any laxative use during the trial; an allergic reaction to Ma'aljobon or its improper consumption; or severely impaired general health.

### 2.4 Recruitment and Participant Monitoring

Participants were recruited using a convenience sampling method. Older adults presenting with symptoms of constipation were examined by a physician and provided with detailed information regarding the study's purpose, follow‐up schedule, and the nature of the intervention. Eligible candidates were then screened and randomized.

Throughout the 4‐week intervention period, treatment adherence and the incidence of adverse events were monitored. At each weekly follow‐up visit, participants' self‐reported adherence to the daily consumption protocol was recorded. They were also systematically questioned about any potential adverse events. All reported events, as well as the reasons for any discontinuation from the study, were documented to assess safety and tolerability. Adverse events were graded using the CTCAE v4.0.

### 2.5 Randomization and Blinding

A computer‐generated random allocation sequence was created by a biostatistician who was not involved in participant recruitment or assessment. Permuted block randomization with a block size of two was used to ensure a 1:1 allocation ratio between the intervention (Ma'aljobon) and control (placebo) groups.

Clinical research staff at the study site formally enrolled participants after confirming their eligibility. Allocation concealment was then maintained by assigning the responsibility of labeling and dispensing the products to the study pharmacist, who had sole access to the randomization sequence. The pharmacist prepared the Ma'aljobon and placebo in identical, opaque, and sequentially numbered containers labeled only with codes “A” or “B”. When a participant was enrolled, the pharmacist assigned the next available number in the sequence, ensuring that the enrolling researchers and clinical staff remained unaware of the upcoming allocation.

This double‐blind design ensured that participants, clinical staff, and researchers were kept blind to the treatment assignments throughout the trial. To assess the success of blinding, participants were asked at the final study visit to guess whether they had received the active treatment or the placebo.

### 2.6 Investigational Product and Placebo

The Ma'aljobon (whey) used in this study was prepared by the laboratory of Tehran University of Medical Sciences, following the production process previously detailed by Mirabzadeh et al. [26]. Briefly, 2000 kg of cow's milk was boiled for 25 min and then cooled to 75°C. The milk solids were separated by adding 260 kg of oxymel and 6 kg of vinegar. The resulting whey solution was then spray‐dried at a rate of 250 L/h, with an inlet temperature of 120°C and an outlet temperature of 50°C [26]. To preserve heat‐sensitive proteins, the final product was also freeze‐dried [27]. The final Ma'aljobon powder contained lactose, α‐lactalbumin, β‐lactoglobulin, immunoglobulins, bovine serum albumin, lactoferrin, branched‐chain amino acids (leucine, isoleucine, and valine), and cysteine.

The placebo was designed to be identical to the Ma'aljobon powder in appearance, taste, and packaging. It was composed of lactose (41.3%), calcium carbonate (13.8%), maltodextrin (11.7%), medicinal salt (1.3%), and sugar (31.90%) and contained none of the active whey protein components. The preparation of both the investigational product and the placebo was overseen by traditional medicine consultants.

### 2.7 Intervention Protocol

Participants in both parallel arms of the study received a 450 g container of either Ma'aljobon or the placebo powder. They were instructed to consume a total of 15 g of the assigned powder daily for 4 weeks, beginning the day after randomization. This was administered in three 5‐g doses, each dissolved in a glass of water and consumed before breakfast, lunch, and dinner. To promote treatment adherence, participants received regular telephone calls or text messages. The use of any laxative products was prohibited for the duration of the trial.

### 2.8 Assessment Tools

#### 2.8.1 Rome III Criteria

FC was defined according to the Rome III criteria, which require the presence of two or more of the specified symptoms over the preceding 3 months. The criteria include: straining during at least 25% of defecations; lumpy or hard stools in at least 25% of defecations; a sensation of incomplete evacuation for at least 25% of defecations; a sensation of anorectal obstruction or blockage for at least 25% of defecations; the use of manual maneuvers to facilitate at least 25% of defecations, such as digital evacuation or support of the pelvic floor; and a frequency of fewer than three defecations per week.

#### 2.8.2 Bristol Stool Form Scale (BSFS)

Stool form, which serves as an indicator of intestinal transit time, was assessed using the 7‐point BSFS. The scale classifies stools into seven distinct categories. Type 1 is defined as separate hard lumps, similar to nuts, that are hard to pass. Type 2 is described as sausage‐shaped but lumpy. Type 3 is like a sausage but with visible cracks on its surface. Type 4 appears like a sausage or snake and is smooth and soft. Type 5 consists of soft blobs with clear‐cut edges that are passed easily. Type 6 is characterized by fluffy pieces with ragged edges, presenting as a mushy or pasty stool. Finally, Type 7 is watery with no solid pieces and is entirely liquid [28]. For the purpose of this study's analysis, these stool types were categorized into three groups: hard stools (types 1 and 2), normal stools (types 3 and 4), and loose stools (types 5, 6, and 7).

#### 2.8.3 Outcomes

The primary outcome of the study was the change in stool consistency from baseline to the end of week 4, as measured by the BSFS. Secondary outcomes included weekly changes in all parameters of FC as defined by the Rome III criteria.

#### 2.8.4 Statistical Analysis

All analyses were conducted on the per‐protocol population, which included participants who completed the full 4‐week study. Baseline sociodemographic characteristics were compared between the intervention and control groups using independent *t*‐tests for continuous variables and *χ* ^2^ tests for categorical variables. Given the ordinal nature of the data from the Rome III questionnaire and the BSFS, the non‐parametric Mann–Whitney–Wilcoxon test was used to compare differences between the groups at baseline and at each weekly follow‐up. The *χ* ^2^ test was used to compare changes in stool consistency categories between the groups. For key outcomes, effect sizes were reported to indicate the magnitude of the treatment effect. A *p* value of less than 0.05 was considered statistically significant. All statistical analyses were conducted using SPSS software, version 23.

#### 2.8.5 Ethical Consideration

This study was conducted in accordance with the ethical principles of the Declaration of Helsinki. The study protocol was approved by the Institutional Review Board of the University of Rehabilitation Sciences and Social Welfare (Ethics Code: IR.USWR.REC.1397.005). All participants provided written informed consent prior to enrollment in the trial. The trial was prospectively registered with the Iranian Registry of Clinical Trials (Registration No. IRCT20180624040216N1).

## 3 Results

### 3.1 Participant Flow and Adherence

**Figure 1:**
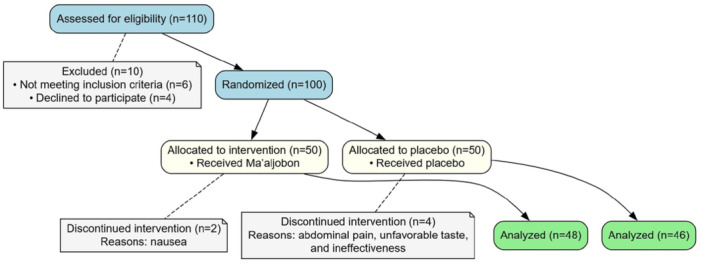
Flow diagram of the participants. CONSORT flow diagram illustrating the progression of participants through the four main stages of the trial: enrollment, allocation, follow‐up, and analysis.

A total of 100 eligible participants were randomized into the study, forming the intention‐to‐treat (ITT) population, with 50 participants allocated to the intervention group and 50 to the control group. Throughout the 4‐week trial, six participants (6%) withdrew from the study, as detailed in the CONSORT flow diagram (Figure 1). Consequently, 94 participants (48 in the intervention group and 46 in the control group) completed the full study protocol and constituted the per‐protocol (PP) population for analysis. Overall treatment adherence was high, with a mean compliance of 99.0% reported in the intervention group and 98.3% in the control group.

### 3.2 Safety and Tolerability

The intervention was generally well‐tolerated, and no serious adverse events were reported in either group during the study period. Of the six participants who withdrew, two from the intervention group cited mild nausea as the reason for discontinuation. In the control group, one participant withdrew due to abdominal pain. The other three withdrawals in the control group were for non‐medical reasons, including dissatisfaction with the placebo's taste and its perceived ineffectiveness.

### 3.3 Baseline Characteristics of the Participants

The analysis was conducted on the 94 participants who completed the study (the per‐protocol population). The sociodemographic characteristics of the intervention and control groups are summarized in Table 1. At baseline, there were no statistically significant differences between the two groups across all examined variables, including age, sex, exercise, chronic diseases, and medication (all *p* > 0.05), indicating that the groups were well‐matched. The study population consisted predominantly of women (73.4%). The mean age was 67.95 ± 7.41 years in the intervention group and 71.82 ± 9.86 years in the control group.

**Table 1:** The sociodemographic characteristics of the participants.

Variable		Intervention group	Control group	*p* value
Number	—	48	46	—
Age	—	67/95 ± 7/41	71/82 ± 9/86	(*p* = 0.21)^a^
Sex	Male	15 (31.3%)	10 (21.7%)	(*p* = 0.30)^b^
	Female	33 (68.7%)	36 (78.3%)	
Exercise	Yes	14 (29.2%)	10 (21.7%)	(*p* = 0.41)^b^
	No	34 (70.8%)	36 (78.3%)	
Chronic disease	Yes	35 (72.9%)	35 (76.1%)	(*p *= 0.59)^b^
	No	13 (27.1%)	11 (23.9%)	
Medication	Yes	39 (81.3%)	35 (76.1%)	(*p* = 0.54)^b^
	No	9 (18.8%)	11 (23.9%)	

^a^ Independent *t*‐test.

^b^ *χ* ^2^ test.

The baseline characteristics related to FC, based on the Rome III criteria, are presented in Table 2. There were no significant differences in any of the FC parameters between the two groups at the start of the study (all *p* > 0.05), confirming their homogeneity in terms of disease severity.

**Table 2:** Baseline constipation characteristics of participants by study group.

Variable	Intervention group (*n *= 48) median (IQR)	Control group (*n* = 46) median (IQR)	*p* value
Straining during defecation	1.5 (1.0–2.0)	2.0 (1.0–2.5)	(*p* = 0.11)^a^
Lumpy or hard stools defecations	2.0 (1.0–3.0)	1.5 (1.0–2.0)	(*p* = 0.38)^a^
Sensation of incomplete evacuation	1.0 (0.0–1.5)	1.0 (0.0–2.0)	(*p* = 0.24)^a^
Sensation of anorectal obstruction/blockage	0.0 (0.0–0.5)	0.0 (0.0–1.0)	(*p *= 0.12)^a^
Manual maneuvers to facilitate	1.0 (0.5–1.5)	1.0 (0.0–2.0)	(*p *= 0.98)^a^
Fewer than three defecations per week	3.0 (2.0–3.0)	2.5 (2.0–3.0)	(*p* = 0.64)^a^
Pain during defecation	1.0 (0.5–1.0)	1.0 (0.0–2.0)	(*p *= 0.61)^a^

Abbreviation: IQR, interquartile range.

^a^ Mann–Whitney–Wilcoxon.

### 3.4 Efficacy of Ma'aljobon on Functional Constipation

The changes in FC parameters from baseline to week 4 are shown in Table 3. At the end of the 4‐week intervention, the Mann–Whitney–Wilcoxon test revealed that the Ma'aljobon group had statistically significant improvements across all measured FC parameters compared to the placebo group (all *p* < 0.001).

**Table 3:** Weekly changes in functional constipation scores between groups.

Variable	Group	Baseline median (IQR)	Week 1 median (IQR)	Week 2 median (IQR)	Week 3 median (IQR)	Week 4 median (IQR)	Week 4 effect size (*r*)
Straining during defecations	Intervention	1.5 (1.0–2.0)	1.0 (0.5–1.5)	0.5 (0.0–1.0)	0.0 (0.0–0.5)	0.0 (0.0–0.0)	0.59
	Control	2.0 (1.0–2.5)	1.5 (1.0–2.0)	1.0 (0.5–1.5)	1.0 (0.0–1.5)	0.7 (0.0–1.0)	
	MWW statistic	912	343	229	381	456	
	*p *value	0.11	< 0.001	< 0.001	< 0.001	< 0.001	
Lumpy or hard stools defecations	Intervention	2.0 (1.0–3.0)	1.5 (1.0–2.0)	1.0 (0.0–1.5)	0.5 (0.0–1.0)	0.0 (0.0–0.0)	0.42
	Control	1.5 (1.0–2.0)	1.5 (1.0–2.0)	1.0 (0.5–2.0)	1.0 (0.0–1.5)	0.5 (0.0–1.0)	
	MWW statistic	996	601	263.50	520.50	644	
	*p* value	0.39	< 0.001	< 0.001	< 0.001	< 0.001	
Sensation of incomplete evacuation	Intervention	1.0 (0.0–1.5)	0.5 (0.0–1.0)	0.0 (0.0–0.5)	0.0 (0.0–0.0)	0.0 (0.0–0.0)	0.41
	Control	1.0 (0.0–2.0)	1.0 (0.0–1.5)	1.0 (0.0–1.5	0.5 (0.0–1.0)	0.4 (0.0–1.0)	
	MWW statistic	967	372	478	599	648	
	*p* value	0.25	< 0.001	< 0.001	< 0.001	< 0.001	
Sensation of anorectal obstruction/blockage	Intervention	0.0 (0.0–0.5)	0.0 (0.0–0.0)	0.0 (0.0–0.0)	0.0 (0.0–0.0)	0.0 (0.0–0.0)	0.24
	Control	0.0 (0.0–1.0)	0.5 (0.0–1.0)	0.5 (0.0–1.0)	0.5 (0.0–1.0)	0.3 (0.0–1.0)	
	MWW statistic	725	616	648	768	840	
	*p* value	0.12	< 0.001	< 0.001	< 0.001	< 0.001	
Manual maneuvers to facilitate defecations	Intervention	1.0 (0.5–1.5)	0.5 (0.0–1.0)	0.0 (0.0–0.5)	0.0 (0.0–0.0)	0.0 (0.0–0.0)	0.22
	Control	1.0 (0.0–2.0)	1.0 (0.0–1.5)	0.5 (0.0–1.0)	0.5 (0.0–1.0)	0.2 (0.0–0.0)	
	MWW statistic	1101	531	597	767	864	
	*p* value	0.94	< 0.001	< 0.001	< 0.001	< 0.001	
Fewer than three defecations per week	Intervention	3.0 (2.0–3.0)	2.0 (1.0–2.5)	1.0 (0.5–1.5)	0.0 (0.0–1.0)	0.0 (0.0–0.0)	0.72
	Control	2.5 (2.0–3.0)	2.0 (1.5–3.0)	1.5 (1.0–2.5)	1.0 (0.5–2.0)	0.7 (0.0–1.0)	
	MWW statistic	1048	488.50	244	287.50	309	
	*p* value	0.65	< 0.001	< 0.001	< 0.001	< 0.001	
Pain during defecation	Intervention	1.0 (0.5–1.0)	0.5 (0.0–1.0)	0.0 (0.0–0.5)	0.0 (0.0–0.0)	0.0 (0.0–0.0)	0.35
	Control	1.0 (0.0–2.0)	1.0 (0.0–1.5)	1.0 (0.0–1.0)	0.5 (0.0–1.0)	0.3 (0.0–1.0)	
	MWW statistic	1079	498	496.50	670.50	720	
	*p* value	0.61	< 0.001	< 0.001	< 0.001	< 0.001	

Abbreviations: IQR, interquartile range; MWW, Mann–Whitney–Wilcoxon.

The effect of the intervention on stool consistency, as measured by the BSFS, is detailed in Table 4. Ma'aljobon demonstrated a significant effect on improving stool consistency (*p* < 0.001). Notably, the number of participants reporting hard stools (types 1 and 2) decreased from 48 to 0 in the intervention group, compared to a decrease from 46 to 13 in the control group.

**Table 4:** Comparison of stool consistency between groups over the 4‐week intervention.

Time point	Group	Hard stool (types 1–2) *n* (%)	Normal stool (types 3–4) *n* (%)	Loose stool (types 5–7) *n* (%)	*p* value
Baseline	Intervention (*n* = 48)	48 (100%)	0 (0%)	0 (0%)	> 0.99^a^
	Control (*n* = 46)	46 (100%)	0 (0%)	0 (0%)	
Week 1	Intervention (*n* = 48)	11 (22.9%)	37 (77.1%)	0 (0%)	< 0.001^a^
	Control (*n* = 46)	38 (82.6%)	8 (17.4%)	0 (0%)	
Week 2	Intervention (*n* = 48)	2 (4.2%)	46 (95.8%)	3 (6.2%)	< 0.001^a^
	Control (*n* = 46)	26 (56.5%)	20 (43.5%)	0 (0%)	
Week 3	Intervention (*n* = 48)	0 (0%)	45 (93.8%)	15 (31.2%)	< 0.001^a^
	Control (*n* = 46)	16 (34.8%)	30 (65.2%)	0 (0%)	
Week 4	Intervention (*n* = 48)	0 (0%)	33 (68.8%)	0 (0%)	< 0.001^a^
	Control (*n* = 46)	13 (28.3%)	33 (71.7%)	0 (0%)	

*Note:* The odds ratio for having normal versus hard stool at Week 4 was significantly higher in the Ma'aljobon group compared to the placebo group (OR = 39.1, 95% CI [2.3, 670.3]).

^a^ *χ* ^2^ test.

## 4 Discussion

The present study, a randomized, double‐blind, placebo‐controlled trial, demonstrated that Ma'aljobon, a traditional whey‐based product, significantly improves the symptoms of FC in older adults. Compared to the placebo group, participants receiving Ma'aljobon experienced statistically significant improvements across all measured parameters of FC, including a notable enhancement in stool consistency. Our findings are consistent with a previous trial by Navabzadeh et al., which showed that Ma'aljobon improved FC in hypertensive patients, although that study was smaller and included a broader age range [29]. Our study builds on this by providing robust evidence for the efficacy of Ma'aljobon specifically within a geriatric population.

A notable characteristic of our sample was the predominance of female participants (73.4%). This distribution, while potentially limiting the generalizability of our findings to elderly men, is consistent with epidemiological data. For example, a major systematic review and meta‐analysis confirmed that chronic constipation is significantly more prevalent in women than in men [30], suggesting our sample reflects the demographic typically affected by this condition. Similarly, while a numerical difference in mean age was observed between the study groups, this difference was not statistically significant, supporting the overall comparability of the groups at baseline.

The considerable improvement in the placebo group is a common finding in trials for functional gastrointestinal disorders [31]. This response can be attributed to a combination of factors, including patient expectations, increased self‐monitoring during the trial, and the composition of our placebo, which contained lactose (41.3%) and may have exerted a mild osmotic laxative effect. Importantly, Ma'aljobon demonstrated a statistically superior efficacy compared to the placebo, confirming a true therapeutic benefit beyond these non‐specific effects.

Our findings align with other research on whey‐based products, even in different populations. For instance, a clinical trial by Xinias et al. demonstrated that an infant formula containing whey hydrolysate significantly improved stool consistency in infants with FC, as measured by the BSFS. Although the study population and specific formulation differ from ours, it provides collateral support for the beneficial effects of whey components on stool form [25].

The therapeutic effects observed in our trial can be understood through a multi‐faceted mechanism that bridges traditional concepts with modern science. In ITM, constipation is often attributed to “dryness” in the intestines, and Ma'aljobon, with its “moisturizing” properties, is believed to counteract this. This traditional concept is consistent with contemporary scientific understanding of osmotic and prebiotic laxative effects.

The primary mechanism is likely the potent prebiotic activity of whey proteins. Components such as α‐lactalbumin have been shown to support *Bifidobacterium* as the predominant organism in gut models while suppressing the growth of less desirable bacteria [23]. Furthermore, other proteins like lactoferrin contribute not merely as a food source for beneficial bacteria, but also through direct antimicrobial action against gut pathogens [24], thereby creating a healthier and more balanced microbial environment. This modulation of the gut microbiota leads to several downstream benefits. The enhanced population of beneficial bacteria ferments the lactose present in whey to produce short‐chain fatty acids (SCFAs), such as acetate, propionate, and butyrate. Butyrate is particularly crucial as it serves as the primary energy source for colonocytes, supporting the health of the intestinal lining [24]. This fermentation process also lowers colonic pH, which both inhibits the growth of pathogenic bacteria and stimulates peristalsis, further reducing colonic transit time. These prebiotic effects work in synergy with a simpler osmotic mechanism, where unabsorbed lactose draws water into the colon, softening stool [24].

Finally, Ma'aljobon's efficacy is attributed to its composition, as it is free of casein—the milk protein fraction implicated in constipation in some individuals [32]—which is removed during the cheese‐making process. This process isolates and concentrates the whey proteins, which are associated with the beneficial prebiotic and pro‐motility effects discussed previously.

## 5 Limitations

This study has several limitations. The generalizability of the findings may be limited as the trial was conducted at a single center using a convenience sampling method and included a modest sample size with a majority of female participants. While the gender distribution may reflect the higher prevalence of constipation in women, results may not be fully applicable to men. Methodologically, our reliance on self‐reported outcomes for symptoms is subject to recall bias, and the study lacked objective physiological measures (e.g., colonic transit time) to corroborate these findings. Furthermore, treatment adherence was also self‐reported, and we did not systematically monitor potential confounding variables such as diet or physical activity. The study's 4‐week duration was not designed to assess long‐term efficacy or symptom relapse after treatment cessation. Finally, our statistical analysis was conducted on the per‐protocol population.

## 6 Conclusion and Future Directions

Ma'aljobon has been shown to significantly improve FC. In addition to other common medications, Ma'aljobon can be a beneficial food option for patients with FC, due to its accessibility, affordability, and absence of adverse effects.

Future research should build upon these promising findings. Longer‐term clinical trials are needed to establish the sustained efficacy and safety of Ma'aljobon and to assess symptom relapse after treatment cessation. Incorporating objective physiological measures, such as colonic transit time, would provide a more robust validation of the observed clinical improvements. Furthermore, studies that include stool sample analysis could directly investigate the proposed prebiotic mechanisms of action. Finally, replicating these results in larger, multi‐center trials is essential to confirm the generalizability of our findings to diverse elderly populations.

## Author Contributions

**Farzaneh Saadat Talab:** conceptualization, project administration, resources, software, supervision. **Mehrdad Karimi:** data curation, investigation, methodology, supervision. **Hadi Najafi:** data curation, formal analysis, methodology, writing – original draft, writing – review and editing. **Atefe Esmailpour Moalem:** data curation, investigation, writing – review and editing. **Shahab Papi:** conceptualization, formal analysis, methodology, project administration, supervision, software.

## Funding

The authors received no specific funding for this study.

## Conflicts of Interest

The authors declare no conflicts of interest.

## Transparency Statement

The corresponding author, Shahab Papi, affirms that this manuscript is an honest, accurate, and transparent account of the study being reported; that no important aspects of the study have been omitted; and that any discrepancies from the study as planned (and, if relevant, registered) have been explained.

## Data Availability

The data that support the findings of this study are available from the corresponding author upon reasonable request.
